# Supplementary figures and images for: An amplified derepression controller with multisite inhibition and positive feedback
Source: PLoS One. 2021 Mar 9;16(3):e0241654. doi: 10.1371/journal.pone.0241654 (PMC7943023; doi:10.1371/journal.pone.0241654)

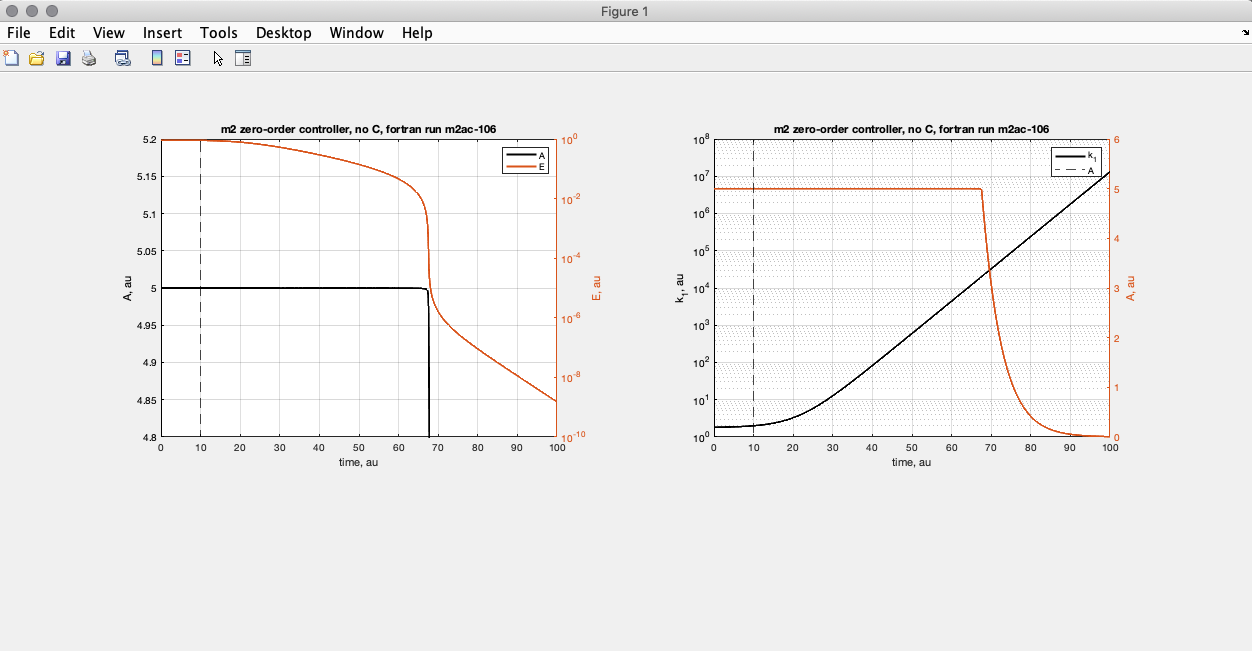

Supplement: S1 Matlab — A zip-file with Matlab programs showing results from Fig 4 (n = 4), Fig 6 (n = 4, i = 1), Figs 8, 10 and 14. (ZIP) [file pone.0241654.s001.zip › S1 Matlab/Fig4 (n=4)/matlab_screenshot_m2ac-106.png]

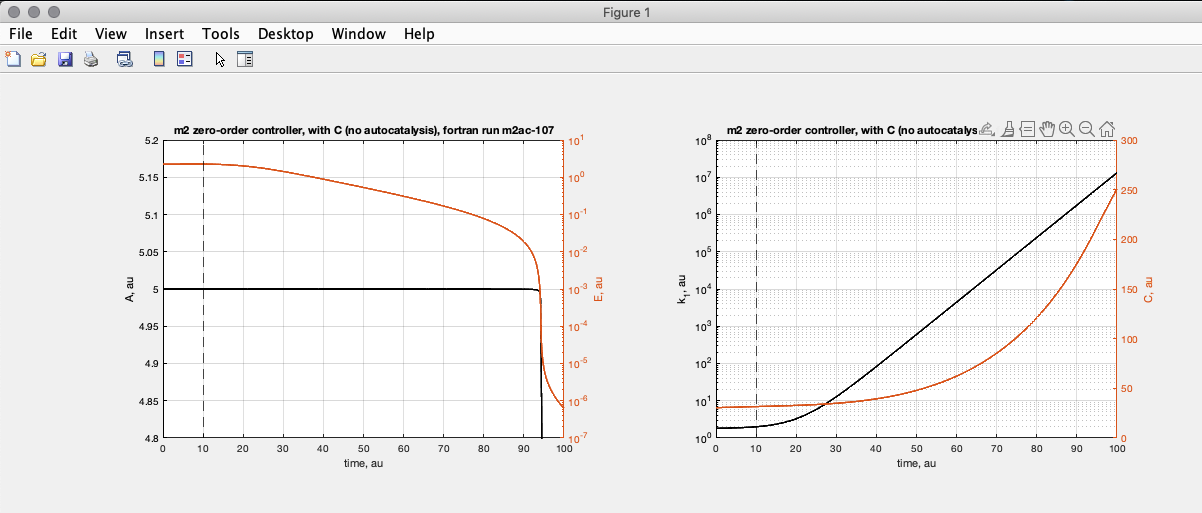

Supplement: S1 Matlab — A zip-file with Matlab programs showing results from Fig 4 (n = 4), Fig 6 (n = 4, i = 1), Figs 8, 10 and 14. (ZIP) [file pone.0241654.s001.zip › S1 Matlab/Fig6 (n=4, i=1)/matlab_screenshot_m2ac-107.png]

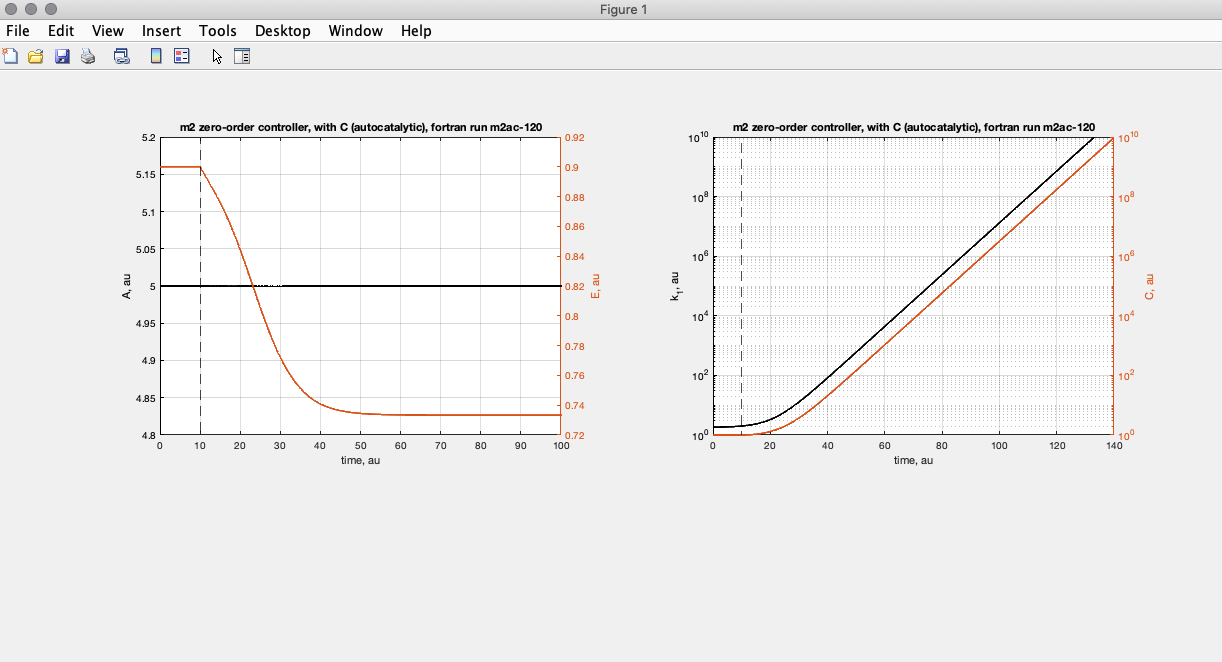

Supplement: S1 Matlab — A zip-file with Matlab programs showing results from Fig 4 (n = 4), Fig 6 (n = 4, i = 1), Figs 8, 10 and 14. (ZIP) [file pone.0241654.s001.zip › S1 Matlab/Fig8/matlab_screenshot_m2ac-120.png]

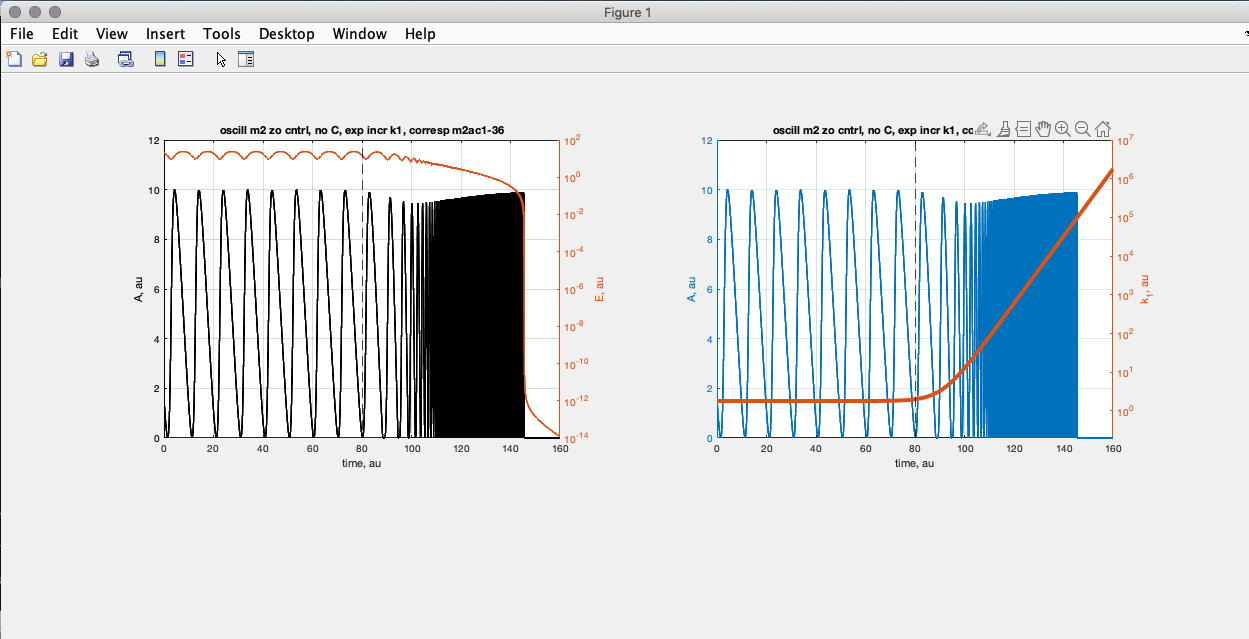

Supplement: S1 Matlab — A zip-file with Matlab programs showing results from Fig 4 (n = 4), Fig 6 (n = 4, i = 1), Figs 8, 10 and 14. (ZIP) [file pone.0241654.s001.zip › S1 Matlab/Fig14/matlab_screenshot_m2ac1-36a.png]

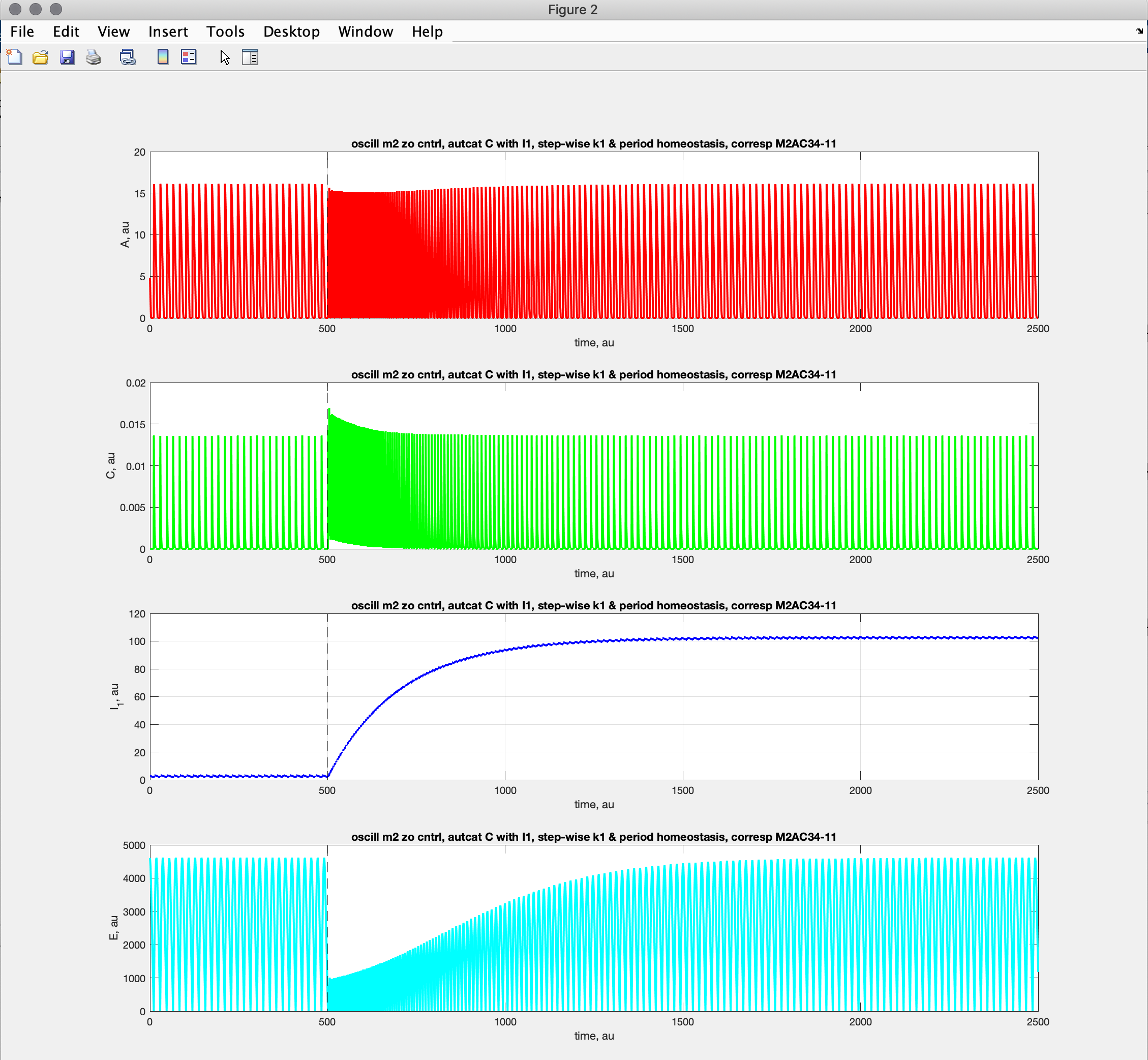

Supplement: S1 Matlab — A zip-file with Matlab programs showing results from Fig 4 (n = 4), Fig 6 (n = 4, i = 1), Figs 8, 10 and 14. (ZIP) [file pone.0241654.s001.zip › S1 Matlab/Fig21/matlab_screenshot_M2AC34-11.png]

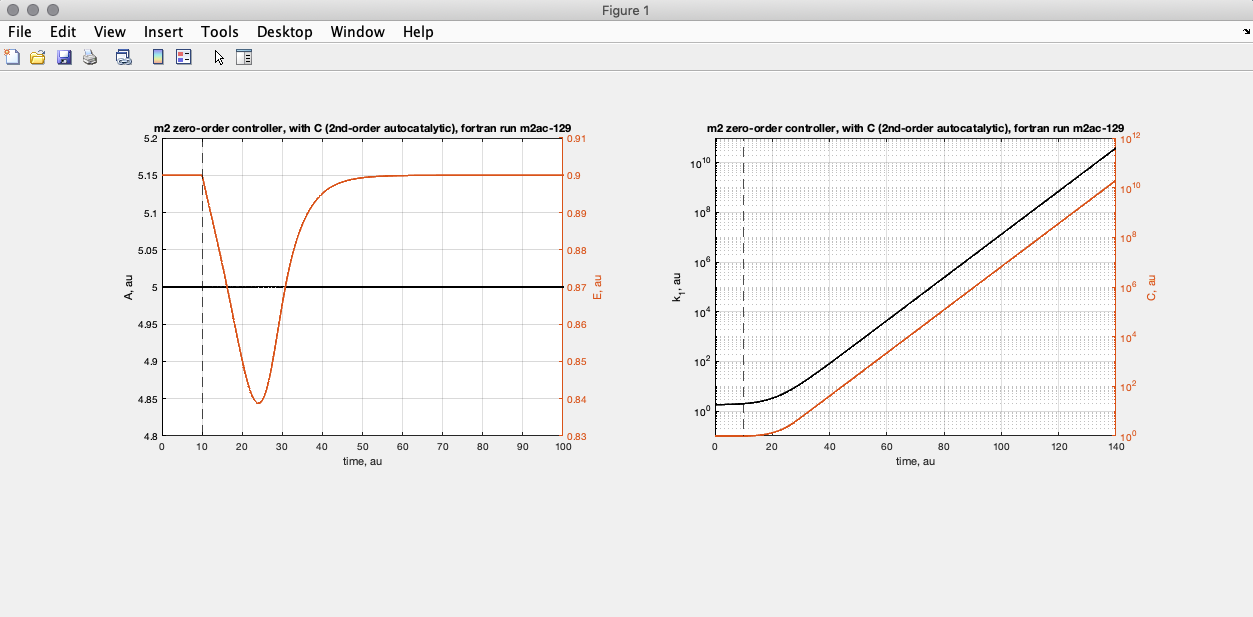

Supplement: S1 Matlab — A zip-file with Matlab programs showing results from Fig 4 (n = 4), Fig 6 (n = 4, i = 1), Figs 8, 10 and 14. (ZIP) [file pone.0241654.s001.zip › S1 Matlab/Fig10/matlab_screenshot_m2ac-129.png]

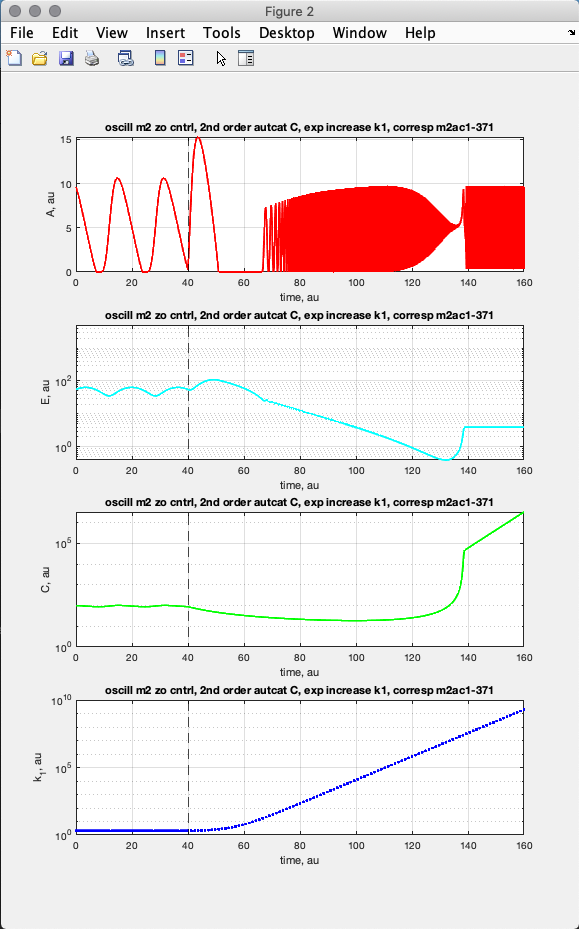

Supplement: S1 Matlab — A zip-file with Matlab programs showing results from Fig 4 (n = 4), Fig 6 (n = 4, i = 1), Figs 8, 10 and 14. (ZIP) [file pone.0241654.s001.zip › S1 Matlab/Fig18/matlab_screenshot_m2ac1-371.png]
