# Supplementary material for: An amplified derepression controller with multisite inhibition and positive feedback
Source: PLoS One. 2021 Mar 9;16(3):e0241654. doi: 10.1371/journal.pone.0241654 (PMC7943023; doi:10.1371/journal.pone.0241654)
Supplement: S1 Text — Comparison between controllers (Fig 7) having first-order and second-order autocatalytic generation of C. (PDF) [file pone.0241654.s002.pdf]

S1 Text

An amplified derepression controller with multisite inhibition and positive feedback

G. Drobac, Q. Waheed, B. Heidari, P. Ruoff*
Department of Chemistry, Bioscience, and Environmental Engineering,
University of Stavanger, Norway

*Corresponding author. Address: Department of Chemistry, Bioscience, and Environmental Engineering, University of Stavanger, Stavanger, Norway, Tel.: (47) 5183-1887, E-mail: peter.ruoff@uis.no

Controller performance towards step-wise changes in k_1 and linearly increasing k_1 values

As shown in the schematic diagram by Fig 7 and Eqs 11 and 12 in the main text, a positive feedback can be implemented by first-order or second-order autocatalysis in C, which increase controller performance in terms of its lifetime.

Here, results with step-wise changes and with linear increase in k_1 are shown when C is produced by first-order and second-order autocatalysis.

First-order autocatalysis in C

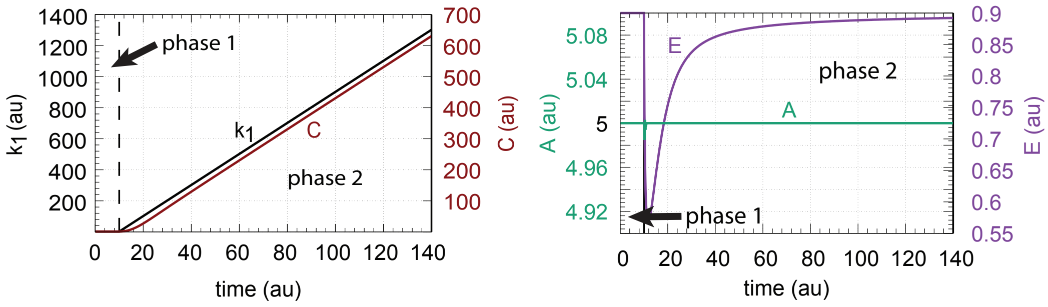

Figure S1: Controller performance with first-order autocatalysis in C (Eq. 11 in the main text) and linear increase of k_1 ($\dot{k}_1$=10.0). Phase 1: the controller is at steady state at its set-point A_{set}=5.0 with constant k_1=2.0. Initial concentrations: A_0=5.0, E_0=0.9, C_0=1.0. Phase 2: k_1 increases linearly, $k_2=1 \times 10^5$, $k_3=5 \times 10^2$, $k_4=1 \times 10^2$, k_5=10.0, and k_6=1.0. $K_M=1 \times 10^{-6}$, K_I=0.1, $n = 4$ (Eq 6 in the main text). Left panel: k_1 and C as a function of time; right panel: A and E as a function of time.

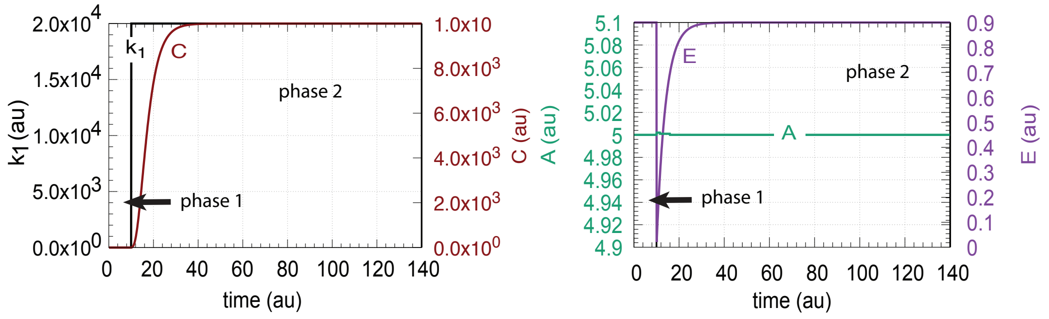

Figure S2: Controller performance with first-order autocatalysis in C (Eq. 11 in the main text) and step-wise increase of k_1. Phase 1: the controller is at steady state at its set-point A_{set}=5.0 with constant k_1=2.0. Initial concentrations: A_0=5.0, E_0=0.9, C_0=1.0. Phase 2: k_1=2×10^4, k_2=1×10^5, k_3=5×10^2, k_4=1×10^2, k_5=10.0, and k_6=1.0. K_M=1×10^{-6}, K_I=0.1, $n = 4$ (Eq 6 in the main text). Left panel: k_1 and C as a function of time; right panel: A and E as a function of time.

Second-order autocatalysis in C

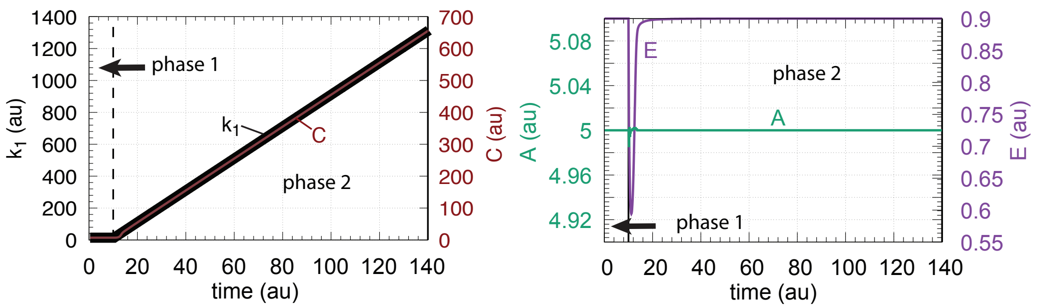

Figure S3: Controller performance with second-order autocatalysis in C (Eq. 12 in the main text) and linear increase of k_1 ($\dot{k}_1$=10.0). Phase 1: the controller is at steady state at its set-point A_{set}=5.0 with constant k_1=2.0. Initial concentrations: A_0=5.0, E_0=0.9, C_0=1.0. Phase 2: k_1 increases linearly, k_2=1×10^5, k_3=5×10^2, k_4=1×10^2, k_5=10.0, and k_6=1.0. K_M=1×10^{-6}, K_I=0.1, $n = 4$ (Eq 6 in the main text). Left panel: k_1 and C as a function of time; right panel: A and E as a function of time.

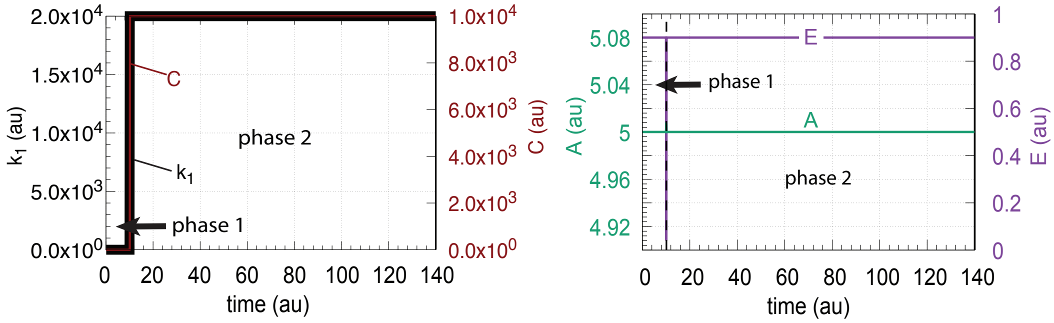

Figure S4: Controller performance with second-order autocatalysis in C (Eq. 12 in the main text) and step-wise increase of k_1. Phase 1: the controller is at steady state at its set-point A_{set}=5.0 with constant k_1=2.0. Initial concentrations: A_0=5.0, E_0=0.9, C_0=1.0. Phase 2: k_1=2×10^4, k_2=1×10^5, k_3=5×10^2, k_4=1×10^2, k_5=10.0, and k_6=1.0. K_M=1×10^{-6}, K_I=0.1, $n=4$ (Eq 6 in the main text). Left panel: k_1 and C as a function of time; right panel: A and E as a function of time.
