# Supplementary material for: An amplified derepression controller with multisite inhibition and positive feedback
Source: PLoS One. 2021 Mar 9;16(3):e0241654. doi: 10.1371/journal.pone.0241654 (PMC7943023; doi:10.1371/journal.pone.0241654)
Supplement: S2 Text — It is shown that autocatalysis with an order larger than one shows hyperbolic growth. (PDF) [file pone.0241654.s003.pdf]

S2 Text

An amplified derepression controller with multisite inhibition and positive feedback

G. Drobac, Q. Waheed, B. Heidari, P. Ruoff*
Department of Chemistry, Bioscience, and Environmental Engineering,
University of Stavanger, Norway

*Corresponding author. Address: Department of Chemistry, Bioscience, and Environmental Engineering, University of Stavanger, Stavanger, Norway, Tel.: (47) 5183-1887, E-mail: peter.ruoff@uis.no

Hyperbolic growth by higher-order autocatalysis

We consider the following autocatalytic reaction with reaction order p>1:

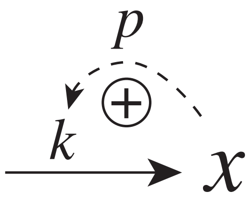

Figure S1: Scheme of an autocatalytic process in x with reaction order p>1.

The rate equation of x is given by

$$\frac{dx}{dt} = k \cdot x^p; \quad p > 1 \tag{S1}$$

By separating the variables

$$\frac{dx}{x^p} = k \cdot dt \implies x^{-p}dx = k \cdot dt \tag{S2}$$

and integration we can get

$$\int_{x_0}^{x(t)} \frac{dx}{x^p} = \int_0^t k \cdot dt = k \cdot t \tag{S3}$$

$$\frac{x^{1-p}}{1-p} - \frac{{x_0}^{1-p}}{1-p} = k \cdot t \tag{S4}$$

with the following expression for x

$$x(t) = \frac{x_0}{(1 - x_0{}^{p-1}(p-1)k \cdot t)^{\frac{1}{p-1}}} \tag{S5}$$

Fig S2 shows x as a function of t when x_0=0.1, p=2, and k=1.0.

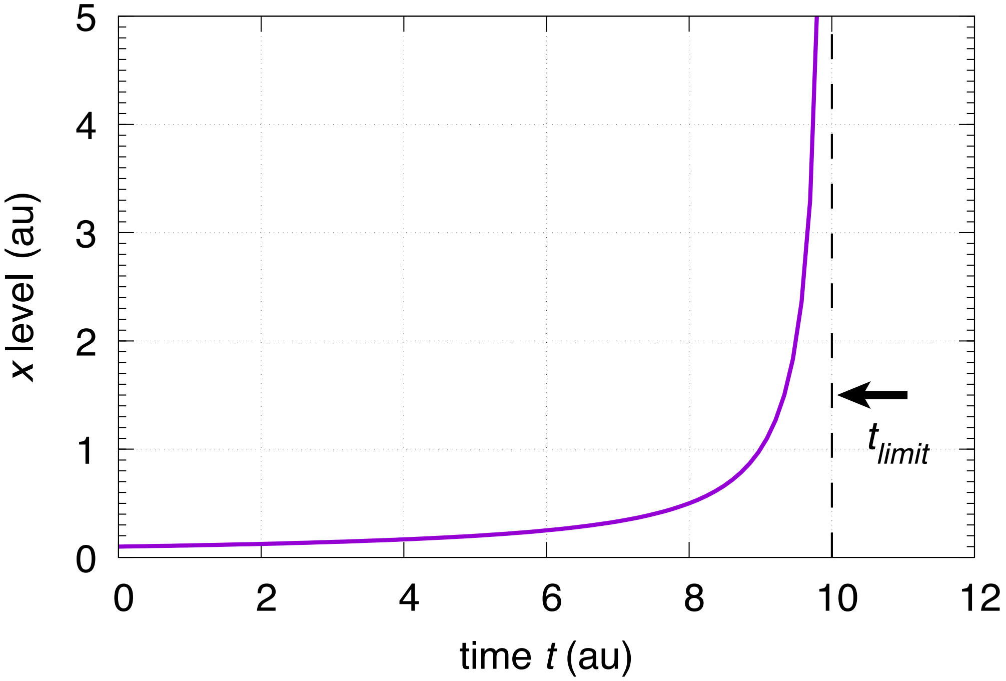

Figure S2: Hyperbolic growth of x when x_0=0.1, p=2, and k=1.0. t_{limit} is the infinity limit, i.e. the time when x reaches infinity.

Doubling time τ and infinity limit for hyperbolic growth

The doubling time τ is the time needed to double the amount/concentration of x. For example, starting with x_0 at a certain time t_0 τ can be found by setting

$$x = 2x(t_0) = 2x_0 \tag{S6}$$

Note that τ is not a constant but a function of the starting x_0 concentration, i.e. τ=$\tau(x_0)$.

Using Eq S5 and the condition for τ (Eq S6)

$$2x_0 = \frac{x_0}{(1 - {x_0}^{p-1}(p-1)k \cdot \tau(x_0))^{\frac{1}{p-1}}} \tag{S7}$$

we get an expression for $\tau(x_0)$ (also termed τ_0)

$$\tau_0 = \tau(x_0) = \frac{2^{p-1} - 1}{2^{p-1}{x_0}^{p-1}(p-1)k} \tag{S8}$$

or alternatively

$$\tau(2x_0) = \tau(x) = \frac{2^{p-1} - 1}{2^{p-1}x^{p-1}(p-1)k} \tag{S9}$$

Observing that k and p are constants, we can simplify Eqs S8 and S9 to respectively

$$\tau_0 = \frac{K}{{x_0}^{p-1}} \quad \text{or} \quad \tau(x) = \frac{K}{x^{p-1}} \tag{S10}$$

where K is a constant.
The infinity limit t_{limit} is calculated by adding successively all the the doubling times τ_0, τ_1, τ_2, etc., by starting with the initial concentration x_0 (see Fig S3), i.e.

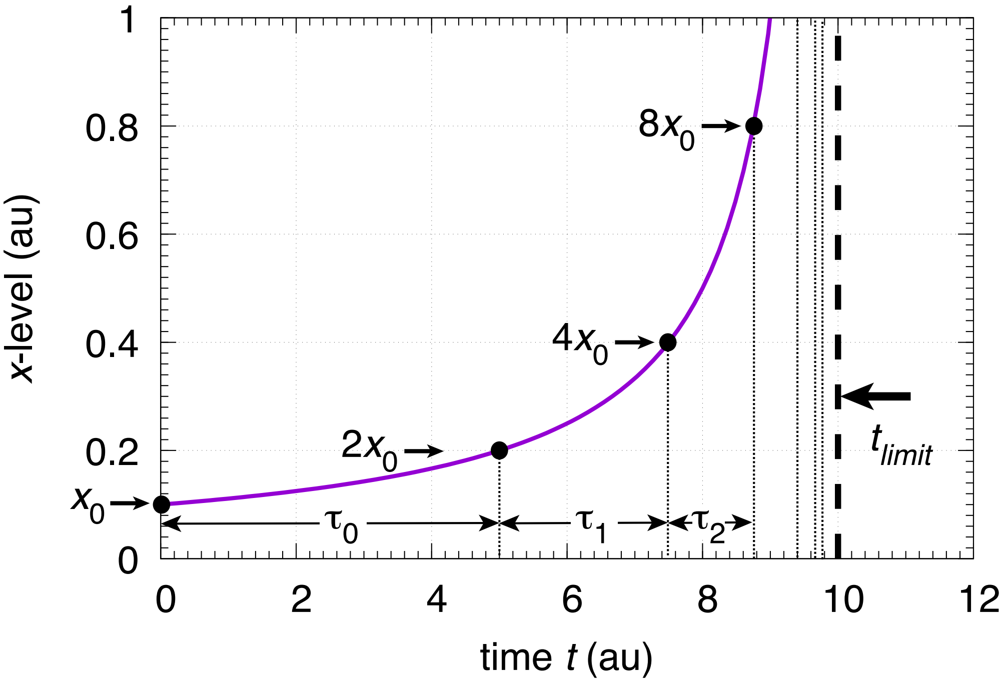

Figure S3: t_{limit} can be calculated by adding the doubling times τ_0, τ_1, τ_2, etc. In this figure we start with x=x_0=0.1. Parameters k and p have the same values as in Fig S2.

$$t_{limit} = \tau_0 + \tau_1 + \tau_2 + ... = \frac{K}{{x_0}^{p-1}} + \frac{K}{2{x_0}^{p-1}} + \frac{K}{4{x_0}^{p-1}} + ... \tag{S11}$$

$$t_{limit} = \frac{K}{{x_0}^{p-1}} \left(1 + \frac{1}{2^{p-1}} + \frac{1}{2^{2(p-1)}} + \frac{1}{2^{3(p-1)}} + ... \right) \tag{S12}$$

$$= \tau_0 \left(1 + (\frac{1}{2^{p-1}})^1 + (\frac{1}{2^{p-1}})^2 + (\frac{1}{2^{p-1}})^3 + ... \right) \tag{S13}$$

$$= \tau_0 \sum_{n=0}^{n=\infty} a^n = \frac{\tau_0}{1-a} \quad \text{with} \quad a = \frac{1}{2^{p-1}} \tag{S14}$$

Eq S14 converges only when a<1, which is equivalent with the condition p>1. Only then hyperbolic growth is observed.
