# Supplementary material for: An amplified derepression controller with multisite inhibition and positive feedback
Source: PLoS One. 2021 Mar 9;16(3):e0241654. doi: 10.1371/journal.pone.0241654 (PMC7943023; doi:10.1371/journal.pone.0241654)
Supplement: Attachment — Submitted filename: review.pdf [file pone.0241654.s004.pdf]

Review

This paper considers an amplified derepression controller that can induce homeostasis on a species in a certain biochemical system against a time-dependent perturbation. This project might be motivated by the fact that the controllability of motif 2 can suffer from breakdown under rapidly increasing external perturbation. In order to either expend the lifetime of the controller or avoid the breakdown, the authors have provided a variety of different control motifs where the target chemical species can admit homeostasis under either exponentially growing perturbations or hyperbolically growing perturbations. In particular, they showed that it is possible for motif 2 system to extend the lifetime of the controller when the controller admits multisite inhibition and an additional variable increasing the compensatory flux. The same control motif is also valid when the target species in motif 2 is oscillating with a zero-order degradation. They showed that not only the time-average of the target species can be homeostatically controlled but also the lifetime of the oscillation can be controlled even under a rapidly growing time-dependent disturbance. Furthermore, it was also shown that with an additional variable I, the period of the oscillation can be maintained almost at the same level within two different phases of a step-wise perturbation.

This manuscript is well-written and very interesting to read. The introduction of the paper provides a nice description of the flow chart of control theory from the era of mechanical control to applications for physiology and biology. It should also be appreciated that the authors introduced different control features (such as multisite inhibition, a new variable C or I, and the positive feedback of C) in a stage-by-stage manner in order to show how the control motif can be improved. They also provide schematic figures and plots that can substantially help readers to easily follow the outline of the paper. Despite the well-elaborated main flow of the paper, more mathematical proof or intuitions need to be provided to explain why such additional control features will be required to achieve the aim of the controller. Therefore I would like to suggest the following major and minor revisions before publication.

Major issues

1. Mathematical proofs or more comprehensive intuitions should be provided at many parts in the paper, especially
   (a) Line 58: Why the breakdown takes place when $E = K_I$??
   (b) Line 60–64: why a lower K_I induces a longer lifespan?
   (c) Eq (8): It is not straightforward to see why Eq (8) is valid.
   (d) Line 140: How can we guarantee that A will stay at the set-point when k_1 grows further? As Fig 3, A can encounter a breakdown if k_1 grows further. Without mathematical

proof or any intuitive descriptions, there is no way to guarantee this. I think this can be shown simply by solving $\dot{E} = 0$ and $\dot{C} = 0$. Then by solving $\dot{A} = 0$, we could prove that A is approximately equal to the set point in the long run.

(e) Line 154: With Fig 8 left panel it seems C is almost k_1+constant. But indeed, $\dot{A} = 0$ in Eq (7) implies that $C = k_1\times$ constant. I think the authors should mention this.

(f) Line 181–183: The derivative of C is not zero for t large enough. Eq (15) is only valid when k_1 is a step-wise change. To address this rigorously, the author should use the fact that $\frac{\dot{C}}{C^2}$ goes to zero as t goes to infinity rather than Eq (10)= 0.

(g) Line 282–286: This part was really interesting to me. If the authors can provide any intuition behind this, that could make this paper better pretty much.

(h) Line 296 and Eq (28): I think the derivative of C never converges to 0 as shown in Fig 21 c. I guess Eq (28) is actually derived by using the fact that $\frac{1}{t}(\ln C(t) + constant) \to 0$ as $t \to \infty$. If so, the authors need to show this.

(i) Eq (28): Why we need to see this quantity? Any meaning?

2. Line 158: I guess Ref [32] significantly influences the main idea of this paper. Hence I wonder what's the key difference or key development of this manuscript in comparison to Ref [32]? I think this should be mentioned in Introduction.

Minor issues

1. Line 30: To my knowledge, the controller defined in [28,29] maintains its controllability even with a step-wise time-dependent transient perturbation on the system parameters.

2. Line 33: This sounds like all the feedback structures that handle time-dependent perturbations well are based on depression kinetics. If the authors do not mean this, this sentence should be toned down.

3. Line 69–71: what does 'control species' mean?

4. Line 94: This implies that for $n = 1$, the graph of E in Fig 4 and the graph of E_3 in Fig 3 must be the same. But they do not look the same.

5. Line 133: It is hard to understand why the author came up with the step-wise changes all of sudden. As long as a steady-state exists, solving $\dot{C} = 0$ implies (11) independently on the step-wise change of k_1.

6. Line 170: Before I see Fig 12, I did not think the system maintains homeostasis for A because A eventually encounters breakdown. So it would better to show Fig 12, especially the plots without C before Figure 9 for a better flow of the paper.

7. Fig 11: How A behaves around 21.249999. Is it oscillating?

8. Eq (16): Does it converges as τ goes to infinity?

9. Line 209: What does ‘certain time interval’ mean?

10. Eq (19): I think the authors should provide a reference for this.

11. Fig 17 (f) does not look an exponential decrease. To show this clearly, the log-scale can be used.

12. Line 244: Why we consider this? Why the paper considers this only for the second-order autocatalysis?

13. Fig 17: It was hard to follow the caption of Fig 17. What’s the difference between green and white outlined $< A >$?

14. Line 308: Should Fig 8 be cited here instead of Fig 9?

15. Line 309: Does this mean if k_1 stops to increase, then the controller breaks down? Why?

16. Line 311–315: I do not follow this part. For making the paper more self-constrained, the authors need to describe this more precisely.

17. Line 320–321: What’s the difference between C-signaling and C? And how this scenario can take place?
