# Supplementary material for: An amplified derepression controller with multisite inhibition and positive feedback
Source: PLoS One. 2021 Mar 9;16(3):e0241654. doi: 10.1371/journal.pone.0241654 (PMC7943023; doi:10.1371/journal.pone.0241654)
Supplement: Attachment — Submitted filename: Response to Reviewers.pdf [file pone.0241654.s005.pdf]

Changes made in the revised manuscript based on the comments by Reviewer #1

Changes made in the manuscript with respect to the comments by Reviewer #1 are indicated in red in the marked-up copy of the revised manuscript.

(1) Reviewer #1: ”Line 35: It seems rather abrupt and sudden to mention motif 2 from Ref [18] without providing proper motivation on why this motif is of particular interest compared to other motifs in Ref [18]. Some discussion would be great.”

Response: *Yes, we agree, the transition to motif 2 appears rather abrupt. We have added the following paragraph, which describes the reasons why this motif has been chosen (lines 33-40):*

”Feedback structures based on derepression kinetics [32] have been found to perform remarkably well when exposed to different time-dependent perturbations. In this study we have focussed on one of the derepression motifs, motif 2 (Ref. [18]). The reasons why we have chosen this motif is, on one hand, its extraordinary capability to compensate against time-dependent perturbations [32,33], and, on the other hand, its frequent occurrence in biochemistry and physiology. For example, the motif occurs repeatedly in the regulation of enzymes [34,35], or has been used as a basic model (the Goodwin model [36]) to describe biological oscillations [37].”

2) Reviewer #1: ”Equation (1) and (2). Here is a suggestion: The authors should consider changing the use of variable E to U to (i) avoid potential associating E with Error and (ii) U is commonly known in control community to represent control action/signal. As a note, it took me quite a while to realise that E is not error but control action, which left me initially quite confused.”

Response: *In our previous work we have kept the term E for the manipulated variable, because the concentration of E is proportional to the integrated error, i.e.,* $E = \int_0^t \epsilon(\tau)d\tau$. *This is the major reason why we wish to keep the E terminology for the manipulated variable in our A-E controller motifs, where A and E denote both the chemical/biochemical species and their concentrations. Another reason is that it appears awkward to refer to our earlier work and to have to explain our change in nomenclature. However, since U is consistently used in control-engineering as a symbol for the manipulated variable, we have included the following changes in the manuscript:*

i) In the legend of Fig 1, we mention that E is the manipulated variable and proportional to the integrated error $\epsilon = A_{set} - A$*:*

”We will show below that the concentration of species E, in control engineering terms called the manipulated variable (and generally assigned the name U), is proportional to the integrated error $\epsilon = A_{set} - A$”.

ii) *On page 3 in the revised manuscript, Eq 2 is extended by Eq 3, showing that* $\dot{E}$ *is proportional to* $(A_{set} - A)$*, i.e.,*

$$\dot{E} = k_4 \cdot A - \frac{k_3 \cdot E}{K_M + E} \cong -k_4 \left(\frac{k_3}{k_4} - A \right) = -k_4 \left(A_{set} - A \right) \tag{3}$$

and that the concentration of E is proportional to the integrated error ϵ.

3) Reviewer #1: ”Line 47. The assumption made here is that $K_M \ll E$. It seems to me that the overall numerical simulation is carried out with $K_M \ll E$ and it gives the impression the mechanism of Motif 2 will work based on this assumption. What if K_M is not significantly smaller than E? How would this affect the overall analysis and conclusion? Has this point being considered as there is no certainty that in practice, $K_M \ll E$ often hold.”

Response: *Yes, $K_M \ll E$ is an ideal situation where the controller can keep A precisely at the set-point A_{set}. When K_M values increase the concentration of A will show increasing offsets from A_{set}, i.e., the controller accuracy is decreased. We described the influence of increasing K_M values on controller accuracy earlier; see Fig S9 in the Supporting Material of Ref. 18.*

To address the reviewer's remark, we have added the following paragraph after the section 'Controller breakdown due to saturation' under the heading: 'Controller accuracy: Influence of K_M', starting line 381:

”The above calculations have been performed under ideal accuracy conditions, i.e., we assumed that the K_M values were always low relative to E ($K_M \ll E$) and I_1 ($K_M \ll I_1$), where the expressions $f_E = E/(K_M+E)$ and $f_{I_1} = I_1/(K_M+I_1)$ are effectively 1. If these conditions are no longer met, due to larger K_M values, f_E and f_{I_1} will be lower than 1, and offsets in A and <C> from their ideal set-point values will be observed. For an illustration how K_M can influence controller accuracy, see Fig S9 in the Supplementary Material of Ref 18.”

4) Reviewer #1: ”Page 9, Line 53. The word “breaks” should be brakes.”
Response: *Thank you! Corrected.*

5) Reviewer #1: ”The overall figure quality needs to be improved. There is element of blurring/smearing making the reading of the figure difficult.”

Response: *Poor figure quality has been a repeated complaint by reviewers on previous submitted PLOS ONE manuscripts. We are not sure why. Our original figures are vector-based pdf files generated by gnuplot and slightly annotated by Adobe Illustrator. Figures were then processed by PACE into TIFF format to meet the technical requirements of the journal, which they passed. For review purposes, we have now included the original pdf-figures in the marked-up copy of the manuscript.*

6) Reviewer #1: ”Figure 3: What determines the choice of parameters for the Equation of k_1 shown in left panel of Figure 3? In practice, how common is k_1 be subjected to this exponential type of perturbation and is it common that k_1 having such large value, in the range up to 10^7.”

Response: *The parameters used to describe the exponential and hyperbolic increase of k_1 in Fig 3 and by*

Eq 14, respectively, are those as used before in References 32 and 33. Although exponential and hyperbolic increases have been found in certain natural systems (for example growth of cell volume and certain viral infections) there is to our understanding not much work which has focussed on time dependent perturbations with respect to robust homeostatic control. In this respect, it is difficult to give here a definite answer to the reviewer.

7) Reviewer #1: "Page 10: Line 89-90: Since you have mentioned this - how would the performance of using different value of K_I be? Has this being investigated? And how different should this value of K_I should be?"

Response: *No, we have not investigated the effect of multisite inhibition in the presence of different inhibition sites/inhibition constants. Chapter 8 of Ref 38 gives a description of this. Roughly, our* $(E/K_I)^n$ *term in Eq 6 will then be replaced by a polynomial of the form:*

$$\frac{E}{K_{I_1}} + \frac{E^2}{K_{I_1}K_{I_2}} + ... + \frac{E^n}{K_{I_1}K_{I_2}...K_{I_n}}$$

where the K_{I_i}*'s are dissociation constants. However, if the inhibitor has cooperative binding, then all terms with less than n factors are generally neglected, which leads to Eq 6 with an average* $K_I = \sqrt[n]{K_{I_1}K_{I_2}...K_{I_n}}$*. Please, see, for example, chapter 8 of Ref 38 about this. With respect to the reviewer's comment we have now slightly reformulated the text and also referred specifically to chapter 8 of Ref 38 (sentence starting line 102):*

"*To make things more straightforward, we assume that one, two, or four molecules of E can bind cooperatively to the enzyme/transporter with the average binding constant* K_I*. In this case, Eq 1 is replaced by (see for example chapter 8 of Ref [39])....".*

8) Reviewer #1: "Page 11, Equation (6), (2) and (7).: This is another suggestion: It is quite odd to label equation this manner where (2) is appearing between (6) and (7). I understand it is the same equation but it is rather odd. Why not just label them as (6)-(8) in a sequential manner?"

Response: *We have now labeled the equations in a sequential manner.*

9) Reviewer #1: "Page 15, Line 225 and also across the article starting from here: What is this m2 controller? It hasn't been defined earlier."

Response: *We have now defined the term 'm2 controller' in the legend of Fig 2.*

10) Reviewer #1: "Page 20, Line 340: Can the author clarifies this statement that RVE8 interacts with LHY/CCA1? I don't think that statement is correct as RVE8 does not interact with LHY/CCA1 as shown in the two articles below. If the authors look at the interaction of plant circadian genes shown in Figure 1

of both respective articles, there is no depiction of RVE8 interacting with LHY/CCA1."

Response: *Thank you for this! Actually, we wanted to change that sentence before submission, but apparently this was forgotten. We have now changed it and cite the two papers the reviewer refers to, in addition to a review paper by McClung; see sentence starting line 412:*

"RVE8 (as a homolog of LHY/CCA1) interacts with the promoters of PRR9, PRR5, TOC1, GI and the EC, which in their turn also have an influence on the plant circadian rhythm [64,68,69]."

Changes made in the revised manuscript based on the comments by Reviewer #2

Changes made in the manuscript with respect to the comments by Reviewer #2 are indicated in blue in the marked-up copy of the revised manuscript.

1 (a) Reviewer #2: "Line 58: Why the breakdown takes place when E = KI ??"

Response: *As E decreases with increasing k_1 the compensatory flux $k_2/(1+\frac{E}{K_I})$ will increase and reach finally its maximum value k_2. As long as $E/K_I \gg 1$ the controller is operative. However, the situation becomes critical when $E/K_I \leq 1$ and k_1 continues to increase. Controller breakdown occurs when the maximum compensatory flux k_2 is reached and k_1 grows further. To* <u>*estimate*</u> *the k_1 value when controller performance becomes critical, k_1^{crit}, we use the condition E/K_I=1. Inserting E/K_I=1 into Eq 1 with $\dot{A}$=0, gives Eq 4.*

We have now used the above argument in the manuscript. See the outline in blue, starting line 64. See also the slight reformulation at the end of Fig 3's legend.

1 (b) Reviewer #2: "Line 60–64: why a lower KI induces a longer lifespan?"

Response: *As seen in Fig 4, lower K_I values have practically no influence on the controller's lifespan, but the aggressiveness of the controller is increased.*

1 (c) Reviewer #2: "Eq (8): It is not straightforward to see why Eq (8) is valid."

Response: *The treatment is analogous to that described in 1(a) above. We have tried to make this more explicit in the manuscript by the following addition, lines 136-141, outlined in blue:*

"...the controller starts to break down upon increasing k_1 values. As for the controller without C (Fig 2, Eq 5) we can estimate a critical k_1 value when breakdown starts by setting E/K_I=1 and inserting it into

Eq 7. Solving for k_1 and noting that A is still at its set-point, gives

$$k_1^{\text{crit}} \approx \frac{k_2 C}{2A_{set}} \tag{10}$$

Fig 6 shows that the controller's lifetime is now dependent on C and the values of the rate constants k_5 and k_6."

1 (d) Reviewer #2: "Line 140: How can we guarantee that A will stay at the set-point when k_1 grows further? As Fig 3, A can encounter a breakdown if k_1 grows further. Without mathematical proof or any intuitive descriptions, there is no way to guarantee this. I think this can be shown simply by solving $\dot{E} = 0$ and $\dot{C} = 0$. Then by solving $\dot{A} = 0$, we could prove that A is approximately equal to the set point in the long run."

Response: *Please note that the condition $\dot{C} = 0$ will only work for step-wise perturbations in k_1. When k_1 increases with time, we have that $\dot{C} > 0$.*

An intuitive description of C's role when k_1 increases with time (linearly, exponentially, or hyperbolically) is based on using appropriate kinetic rate laws in generating C, which allows C to follow and compensate the increase in k_1. There is a kind of hierarchical kinetic order, by which time-dependent increases/perturbations of k_1 can be ruled by a controller. We have:

- *hyperbolic controller kinetics (second-order autocatalysis) can compensate first-order autocatalytic growth*
- *first-order autocatalytic controller kinetics can compensate linear growth*

If perturbations have linear, autocatalytic (first-order), or hyperbolic kinetics, then respectively, linear, autocatalytic, or hyperbolic controller kinetics can also oppose the perturbations, but for some controller types an offset, i.e., a deviation from the set-point is observed. This type of kinetic hierarchy has been described and applied in Ref 32.

To address the reviewer's point for an intuitive description we have included the following paragraph (lines 161-176):

"To get an intuitive understanding about the role of C and the required kinetics to oppose different rate laws of k_1, we note that linear and first-order autocatalytic growth rates in k_1 can be compensated by second-order autocatalytic (hyperbolic) generation of C. In other words, compensatory growth based on second-order autocatalysis will dominate over perturbative first-order autocatalytic or linear growth and thereby control it. The reason for this, is because second-order autocatalysis is eventually more rapid than first-order autocatalysis and linear growth. Also, a linear increase of k_1 can be opposed when the compensatory flux is based on first-order autocatalysis, because first-order autocatalysis will in the end become more rapid than linear growth. Ref 32 gives a more detailed description of how such hierarchies between rate laws has been applied with respect to to different time-dependent perturbations and controller motifs.
Thus, the role of C, which is generated by an appropriate rate law, is to be "ahead" of the time-dependent perturbation and oppose it by its influence to the compensatory flux. As a result, E will go into a steady

state and A can be kept, due to Eq 8 and due to the zero-order removal of E, at its homeostatic set-point. Indeed, $\dot{A}$=0 in Eq 7 and constant E implies that C=k_1×constant."

1 (e) Reviewer #2: "Line 154: With Fig 8 left panel it seems C is almost k1+constant. But indeed, $\dot{A}$ = 0 in Eq (7) implies that C = k1 × constant. I think the authors should mention this."

Response: *Thank you for pointing out the implication for C from $\dot{A}$=0. We have now mentioned this in the last sentence starting line 175:*

"Indeed, $\dot{A}$=0 in Eq 7 and constant E implies that C=k_1×constant.".

1 (f) Reviewer #2: "Line 181–183: The derivative of C is not zero for t large enough. Eq (15) is only valid when k1 is a step-wise change. To address this rigorously, the author should use the fact that $\frac{\dot{C}}{C^2}$ goes to zero as t goes to infinity rather than Eq(10)=0."

Response: *Actually, we have been using this fact. We have now reworded the sentence in the revised manuscript to (starting line 213):*

"E's set-point can be calculated by dividing Eq 12 with C^2 and setting the resulting expression to zero, which leads to

$$E_{set} = K_I \left(\frac{k_5}{k_6} - 1 \right) \quad (17)$$

1 (g) Reviewer #2: "Line 282–286: This part was really interesting to me. If the authors can provide any intuition behind this, that could make this paper better pretty much."

Response: *We have added a paragraph (starting line 312) outlining the idea that period homeostasis can be achieved by mechanisms which keep the flux through the oscillator constantly regulated/balanced*:

"In more general terms, a mechanism for period homeostasis can apparently be based on the idea to keep the overall (and frequency determining) flux through the entire oscillator constant, either by the addition of a new controller variable [52], or by keeping all period/flux control coefficients balanced [58,59]. Here we show that period homeostasis can be achieved in a A−E−C oscillator by adding a flux-compensating controller variable to the system. As an example, we use the oscillatory controller described in Fig 15 (including autocatalytic formation of C)."

1 (h) Reviewer #2: "Line 296 and Eq (28): I think the derivative of C never converges to 0 as shown in Fig 21 c. I guess Eq (28) is actually derived by using the fact that $\frac{1}{t}(\ln C\ (t) + \text{constant}) \to 0$ as t → ∞. If so, the authors need to show this."

Response: *Correct, but please note that $<C>$=0.001=constant and that $<\dot{C}>$=0. The derivation of*

$\left\langle \frac{K_I}{K_I+E} \right\rangle = \frac{k_6}{k_5}$ *=0.01 (Eq 30 in revised ms) is based on* $<\dot{C}>=0$ *(Eq 25). Eq 25 is then divided by C leading to Eq 30. Eq 30 is in agreement with the numerical calculations shown in Fig 21.*

1 (i) Reviewer #2: "Eq (28): Why we need to see this quantity? Any meaning?"

Response: *The quantity* $\left\langle \frac{K_I}{K_I+E} \right\rangle = \frac{k_6}{k_5}$ *=0.01 (now Eq 30) is conserved and we looked for an analytical solution/expression for* $<E>$*. However, as we mention in the ms we were not able to find one, and therefore used the numerical solution of* $< E >$*.*

2 Reviewer #2: "Line 158: I guess Ref [32] significantly influences the main idea of this paper. Hence I wonder what's the key difference or key development of this manuscript in comparison to Ref [32]? I think this should be mentioned in Introduction."

Response: *Yes, this is in agreement with the comment by Reviewer #1, no. 1 above, that our motivation for this work should be stated more clearly. Please, see our response to 1) Reviewer #1 above and the red-outlined paragraph in the revised manuscript starting at line 33.*

Minor issues

1. Reviewer #2: "Line 30: To my knowledge, the controller defined in [28,29] maintains its controllability even with a step-wise time-dependent transient perturbation on the system parameters."

Response: *We do not disagree in what the reviewer writes, although it is not quite clear to us to what the reviewer is pointing at. However, to avoid misunderstanding with respect to Refs 28,29, we have in the sentence which starts on line 29 removed the word* *<u>these</u>*. *The sentence reads now:*

"When feedback motifs were investigated towards time-dependent perturbations, it turned out that controller performances can differ significantly, either due to the structure of the feedback loop or due to the kinetics of how the integral controller is implemented [32,33]."

2. Reviewer #2: "Line 33: This sounds like all the feedback structures that handle time-dependent perturbations well are based on depression kinetics. If the authors do not mean this, this sentence should be toned down."

Response: *No, this is not what we meant. With respect to our response to Reviewer #1, no. 1 above, we have rewritten this part. Please, see the red-outlined paragraph in the revised manuscript (line 33 to line 40).*

3. Reviewer #2: "Line 69–71: what does 'control species' mean?"

Response: *We refer here to the controller variable E. We have replaced 'control species' by 'controller variable E; please see line 82 in revised ms.*

4. Reviewer #2: "Line 94: This implies that for n=1, the graph of E in Fig 4 and the graph of E_3 in Fig3 must be the same. But they do not look the same."

Response: *We looked at the original data for these two figures; E in Fig 4 for n=1 and E_3 in Fig 3 show precisely the same time profiles when plotted using the same ordinate scale for E. Please note that the ordinate scales for E in Fig 3 and Fig 4 are different.*

5. Reviewer #2: "Line 133: It is hard to understand why the author came up with the step-wise changes all of sudden. As long as a steady-state exists, solving $\dot{C} = 0$ implies (11) independently on the step-wise change of k_1."

Response: *Yes, the reviewer is correct, as long as a steady state exists, $\dot{C} = 0$ implies (11) (now Eq 13). We have reworded that sentence in this way (starting line 149), but referred to a calculation where A is under homeostatic control, but E is not:*

"Note that for any changes in k_1, as long as a steady state exists, E becomes homeostatic controlled in addition to A, because the conditions $\dot{C}/C$=0 or $\dot{C}/C^2$=0 in respectively Eqs 11 or 12 imply that

$$E_{ss} = \left(\frac{k_5}{k_6} - 1\right) K_I \tag{13}$$

independent of the perturbation k_1. However, a situation where A is homeostatic controlled, but E is not, is given below, when a controller with first-order autocatalysis in C meets a hyperbolically increasing k_1."

6. Reviewer #2: "Line 170: Before I see Fig 12, I did not think the system maintains homeostasis for A because A eventually encounters breakdown. So it would better to show Fig 12, especially the plots without C before Figure 9 for a better flow of the paper."

Response: *In the Introduction we mention (line 43) that motif 2 (m2, Fig 2) can balance exponentially, even hyperbolically increasing perturbations, and refer to [32]. As an example, we show in Fig 3 the case when m2 opposes exponentially increasing k_1. When C has been introduced, we asked the question how well does m2 without C behave in comparison with controllers that have first- or second-order autocatalysis in C? Fig 12 is the answer to that. We feel that this flow appears (to us) more natural than showing the comparison first. So, we would like to keep the order of the figures.*

7. Reviewer #2: "Fig 11: How A behaves around 21.249999. Is it oscillating?"

Response: *When we come close to the '21.25-infinity limit' at t=21.249997, LSODE (the solver) becomes irregular, not oscillatory. At time t=21.249998, LSODE can't meet absolute and relative tolerances any more (which are* 1×10^{-12} *and* 1×10^{-9}*, respectively). We stopped with the calculations at that point.*

8. Reviewer #2: "Eq (16): Does it converges as τ goes to infinity?"

Response: *Our calculations here and in Ref 48 indicate that calculated <A> defined by Eq 18 (revised ms) converges as* τ *becomes large enough (as a practical rule 10 cycles are often sufficient). For example, for regular, sustained oscillations the ratio between integrated A during one period and the period length is a constant, such that Eq 18 converges as* τ *goes to infinity. As an additional remark, Eq 18 even holds when oscillations become chaotic, i.e., both amplitudes and periods show chaotic behavior. See Eqs 20/21 in Thorsen et al., Physica D, 2019, 393: 38-46. https://doi.org/10.1016/j.physd.2019.01.002*

9. Reviewer #2: "Line 209: What does 'certain time interval' mean?"

Response: *By that we meant the simulation and integration times in the examples that follow. To indicate this we have added '(see the examples below)' in line 241 (outlined in blue).*

10. Reviewer #2: "Eq (19): I think the authors should provide a reference for this."

Response: *Done! Please, see line 252.*

11. Reviewer #2: "Fig 17 (f) does not look an exponential decrease. To show this clearly, the log-scale can be used."

Response: *The ordinate of Fig 17f has a log scale.*

12. Reviewer #2: "Line 244: Why we consider this? Why the paper considers this only for the second-order autocatalysis?"

Response: *In order to get a homeostatic response and to avoid overcompensation, we found that when C is generated by second-order autocatalysis we sometimes had to adjust the* k_2 *and* k_5 *values that were used by first-order autocatalysis. We have now changed that part slightly to (see also changed parts outlined in blue in revised ms, starting line 275):*

"When C is generated by second-order autocatalysis (Eq 12) the resulting controller is, as for first-order autocatalysis, able to defend A_{set}*, but values for* k_2 *and* k_5 *had to be adjusted to get a homeostatic response and to avoid overcompensation. Fig 18 shows the case when* k_1 *increases exponentially. We found that an*

increase of k_2 by one order of magnitude during phase 2 was necessary to get a homeostatic response. To avoid overcompensation..."

13. Reviewer #2: Fig 17: It was hard to follow the caption of Fig 17. What's the difference between green and white outlined <*A*>?

Response: *Green <A> is the overall calculated <A> from t=0, while white outlined <A> is the <A> value calculated from t=120 (white vertical line) to t=180, when the controller has taken control. We have now slightly changed this part of the legend to:*

"...(a) A (in purple) and overall <A> (in green, starting from t=0) as a function of time. The white outlined <A> is the <A> value calculated from t=120 (white vertical line) to t=180 time units, showing that for this time region when control is achieved, we have <A>=A_{set}=5.0...."

14. Reviewer #2: "Line 308: Should Fig 8 be cited here instead of Fig 9?"

Response: *Yes, correct; thank you for spotting this! Corrected.*

15. Reviewer #2: "Line 309: Does this mean if k_1 stops to increase, then the controller breaks down? Why?"

Response: *Sorry, that sentence was not clearly written. To avoid misunderstanding, we have slightly changed this paragraph to (see lines 350-356):*

"...Taking the controllers with a first-order autocatalysis in C as an example: although they are clearly able to follow exponentially increasing k_1 values (Figs 8 and 17), their kinetics need to be considered as idealizations. In reality, for example in a cell, reactions are catalyzed by enzymes, reaction components undergo transport, and growth of k_1 and C cannot continue forever. Growth of k_1 and C will eventually encounter limitations, for example due to capacity limits in transport or in enzymatic activity, such that k_1 and/or C may reach a more or less constant upper limit level..... "

16. Reviewer #2: "Line 311–315: I do not follow this part. For making the paper more self-constrained, the authors need to describe this more precisely."

Response: *We have rewritten this section. Please, see the text outlined in blue, lines 342-379.*

17. Reviewer #2: "Line 320–321: What's the difference between C-signaling and C? And how this scenario can take place?"

Response: *In the rewritten section "Controller breakdown due to saturation" we have now included a description of the considered signaling from C to the compensatory flux (starting line 360):*

"Finally, saturation may also occur in the various signal-transduction pathways indicated in the above schemes by dashed lines. There are many, often complex ways how signal transduction occurs in biological systems [61], which we, for the sake of simplicity, have described without any intermediate components."

Ref. 61 gives an overview of the molecular mechanisms in signal transduction.

Additional changes

1.) We have changed the file naming for the Supporting Information according to the PLOS ONE guideline.

2.) In the legend of Fig 16 we have chosen to remove the sentence: "*Controller breakdown occurs just after 160 time units (data not shown)*." This sentence is not essential for the figure.

3.) Lines 186-188: The description of deriving Eq 15 has been changed slightly (outlined in green).
