# Supplementary material for: An amplified derepression controller with multisite inhibition and positive feedback
Source: PLoS One. 2021 Mar 9;16(3):e0241654. doi: 10.1371/journal.pone.0241654 (PMC7943023; doi:10.1371/journal.pone.0241654)
Supplement: Attachment — Submitted filename: review2.pdf [file pone.0241654.s006.pdf]

Review

Major issues

1 (a) Line 58: Why the breakdown takes place when $E = K_I$??

Okay, but I am still curious about why the controller breaks down when $E/K_I \ll 1$. I think the breakdown of the controller is actually up to the size of $k_1(t)$, not the value of E/K_I (of course when k_1 is big, E is small. But what I mean is, the main reason of the breakdown is k_1, not E/K_I in the sense of math). Am I missing something? Also, I do not understand why $E/K_I = 1$ is critical for the controller. How the value 1 came up with? Is it just because we regard that $E/K_I \leq 1$ is small and $E/K_I > 1$ is big? If so, I think this analysis is somewhat crude unless the authors provide more rigorous verification about why the controller becomes critical when $E/K_I = 1$.

1 (h) Line 296 and Eq (28): I think the derivative of C never converges to 0 as shown in Fig 21 c. I guess Eq (28) is actually derived by using the fact that $\frac{1}{t}(\ln C(t) + constant) \to 0$ as $t \to \infty$. If so, the authors need to show this.

To me, it is not obvious how we can derive Eq 30 with $<\dot{C}> = 0$. This condition ($<\dot{C}> = 0$) may not solely imply Eq 30. Suppose $C(t) = e^{-t}$ for which $<\dot{C}> = 0$ when $t \to \infty$. Then

$$\frac{1}{t}\int_0^t \frac{\dot{C}(\tau)}{C(\tau)} d\tau = \frac{1}{t}\int_0^t \frac{K_I}{E + K_I} d\tau.$$

But, since

$$\frac{1}{t}\int_0^t \frac{\dot{C}(\tau)}{C(\tau)} d\tau = \frac{1}{t}\left(\ln C(t) - \ln C(0)\right) \to -1, \quad \text{as } t \to \infty.$$

Is C bounded away from c_0 for some $c_0 > 0$? (it is bounded above as shown in Figure 21) Then the limit $\lim_{t\to\infty} \frac{1}{t}\int_0^t \frac{\dot{C}(\tau)}{C(\tau)} d\tau$ is zero obviously. I think the authors should provide a more rigorous explanation for this.

Minor issues

11 Fig 17 (f) does not look like an exponential decrease. To show this clearly, the log-scale can be used.

If the y-axis in Figure 17 f is in log-scale, it should be indicated in the figure.
